# The global health and care worker compact: evidence base and policy considerations

**DOI:** 10.1136/bmjgh-2023-012337

**Published:** 2023-07-25

**Authors:** Eric A Friedman, Robert Bickford, Charles Bjork, James Campbell, Giorgio Cometto, Alexandra Finch, Catherine Kane, Sarah Wetter, Lawrence Gostin

**Affiliations:** 1 O’Neill Institute for National and Global Health Law, Georgetown University Law Center, Washington, District of Columbia, USA; 2 Edward Bennett Williams Law Library, Georgetown University Law Center, Washington, District of Columbia, USA; 3 Health Workforce Department, World Health Organization, Geneva, Switzerland

**Keywords:** Health policy, Health systems

## Abstract

**Background:**

During the COVID-19 pandemic, and recognising the sacrifice of health and care workers alongside discrimination, violence, poor working conditions and other violations of their rights, health and safety, in 2021 the World Health Assembly requested WHO to develop a global health and care worker compact, building on existing normative documentation, to provide guidance to ‘protect health and care workers and safeguard their rights’.

**Methods:**

A review of existing international law and other normative documents was conducted. We manually searched five main sets of international instruments: (1) International Labour Organization conventions and recommendations; (2) WHO documents; (3) United Nations (UN) human rights treaties and related documents; (4) UN Security Council and General Assembly resolutions and (5) the Geneva Conventions and Additional Protocols. We included only legal or other normative documents with a global or regional focus directly addressing or relevant to health and care workers or workers overall.

**Results:**

More than 70 documents met our search criteria. Collectively, they fell into four domains, within which we identified 10 distinct areas: (1) preventing harm, encompassing (A) occupational hazards, (B) violence and harassment and (C) attacks in situations of fragility, conflict and violence; (2) inclusivity, encompassing (A) non-discrimination and equality; (3) providing support, encompassing (A) fair and equitable remuneration, (B) social protection and (C) enabling work environments and (4) safeguarding rights, encompassing (A) freedom of association and collective bargaining and (B) whistle-blower protections and freedom from retaliation.

**Discussion:**

A robust legal and policy framework exists for supporting health and care workers and safeguarding their rights. Specific human rights, the right to health overall, and other binding and non-binding legal documents provide firm grounding for the compact.

However, these existing commitments are not being fully met. Implementing the compact will require more effective governance mechanisms and new policies, in partnership with health and care workers themselves.

**WHAT IS ALREADY KNOWN ON THIS TOPIC**

- Internationally binding laws, as well as non-binding resolutions and other international instruments, exist through the WHO, International Labour Organization and other international bodies that implicate the rights and well-being of health and care workers.
- A comprehensive collation of what laws and other international instruments exist that are relevant to the rights and well-being of health and care workers, as well as the mix of legally binding and non-binding legal instruments, did not previous exist.

**WHAT THIS STUDY ADDS**

- This study represents the first comprehensive review of international legal and policy instruments that provide for health and care workers’ rights and well-being, information that is necessary to understand the full range of states’ obligations and responsibilities, both binding and non-binding, to their health and care workers. We now know that states have binding and non-binding obligations and responsibilities that underlie the full range of issues that the global health and care workers compact was meant to and did in fact address, and that these obligations encompass the full range of health and care workers’ rights. While each of these obligations is known individually, this synthesis was necessary to develop and understand the nature of the compact. We now know that though the compact is itself non-binding, underlying it throughout are existing obligations, many of which are binding.

**HOW THIS STUDY MIGHT AFFECT RESEARCH, PRACTICE OR POLICY**

- Given that the global health and care worker compact’s provisions are underpinned by existing state obligations and responsibilities, developing new and strengthening existing mechanisms to enable and ensure that states carry out these obligations and responsibilities will be critical for compact implementation and securing the rights and well-being of health and care workers—and consequently, of countries’ entire populations.

## Background

Health and care workers, the majority of whom are women, are central to attaining international and national health.^1 2^ Yet many countries still underinvest in their education and working conditions. The pivotal role of health and care workers during the COVID-19 pandemic, along with the health and psychological impacts they endured in the course of their work, has placed a spotlight on their needs: a renewed and sustained focus on their occupational health and safety; fair remuneration as part of decent working conditions; protection from harassment, workplace violence (which affects all health and care workers, but can be particularly harmful for women), stigma, and discrimination; an enabling work environment with needed resources to perform their roles and; addressing the growing burden of unpaid care work, especially for women, in the health and social care sectors.

Estimates suggest between 80 000 and 180 000 deaths globally among health and care workers due to COVID-19 between January 2020 and May 2021,^3^ with shortage of and inadequate personal protective equipment (PPE) recognised as a key contributing cause.^4^ Many health and care workers lacked access to testing, paid sick time, treatment, and COVID-19 vaccines—even as wealthier countries were vaccinating populations at far lower risk of contracting the virus.^5^ Health and care workers experienced mental health issues during COVID-19 due to a range of causes, from witnessing illness and death at significantly higher rates to experiencing bullying and harassment, and from working extended hours to worrying about their own higher exposure to the virus. They often lacked access to necessary services and support.^6^

Inadequate resources and staffing during the pandemic spurred collective action among health and care workers for safer working conditions.^7^ However, some workers were prevented from or penalised for organising,^8^ while others faced significant legal and practical obstacles.^9^ Of grave concern, some health workers were investigated, detained, and imprisoned for informing the public or other health workers of the scope of the spread of COVID-19,^10^ questioning or criticising their government’ response,^11–13^ and disputing official infection numbers.^13 14^

Violence against health and care workers increased during the COVID-19 pandemic, both in the workplace and in the community due to their occupation.^15–17^

While some of these fundamental workplace challenges have been exacerbated by the pandemic, they existed before. Often, health and care workers who experience workplace violence lack outlets to report it, or do not report it due to fear of retaliation.^18^

Violence against health workers is particularly rife during armed conflicts, with documentation of hundreds having been killed in conflict-affected areas^19^ as well as in other chronic complex emergencies.^20 21^

Health workers have also experienced human rights violations in conflict settings in retaliation against their acting in accordance with their professional and humanitarian ethics, such as providing care to anti-government protesters and other civilians.^22^ Providing impartial care to the sick and wounded on the battlefield is one of the foundations of the Geneva Conventions and has been a humanitarian norm since the mid-19th century. National counterterrorism laws increasingly have been used to prosecute health workers for performing their duties impartially. In at least 10 of 16 countries that have such laws that one study surveyed, counterterrorism legislation has been interpreted to include providing medical care as a form of supporting terrorists.^23 24^

Further, health and care workers face gender-based violence and harassment and other forms of discrimination in the workplace. Though comprising 67% of the global health and care workforce, women hold only 25% of senior roles^25^ and perform the majority (76%) of unpaid care work.^9^ Migrant and ethnic-minority nurses are at higher risk of work-related discrimination than native or ethnic-majority nurses, and discrimination is a leading cause of impaired health among these workers.^9^

Compensation is one area where gender inequality surfaces: Using weighted global estimates, the gender pay gap in the health and care sector ranges from about 15% (in the case of median hourly wages) to about 24% (in the case of mean monthly earnings), and men are over-represented in higher-paid occupations.^26^ More than one-third of the gender pay gap cannot be explained by different working hours and occupational categories.^27 28^ It has been estimated that women contribute US$3–US$4.5 trillion annually to global health, one-third to one-half through unpaid care work.^27 29^ Community health workers, disproportionately women, are frequently underpaid or unpaid.^9 30^

Health and care workers face other compensation inequities. As a human capital-intensive sector, health and care has an over-representation of low-paid workers.^28^ This includes low wages in the long-term care sector, particularly for personal carers. In Organisation for Economic Co-operation and Development (OECD) countries, pay is 35% lower in the long-term care sector than the hospital sector for workers in the same occupation.^31^ Young and newly qualified health and care workers often must perform unpaid or underpaid work during their education and early career.^32^ During the COVID-19 pandemic, students were asked to temporarily delay their education to take on unpaid or underpaid and undersupervised roles.

Health and care workers often lack social protections, especially those working in private homes and those in part-time or temporary work arrangements or with casual contracts.^9 33^ Lack of paid parental and family leave is a particular concern,^34^ while those providing unpaid care often lack access to social benefits more broadly.^9 33^ Furthermore, health and care workers frequently lack enabling work environments,^9 35^ while groups experiencing marginalisation, such as women and migrants, often occupy lower-level positions with little voice in decision-making, which may lead to lack of supportive work environments.^36^

In recognition of the unprecedented difficulties health and care workers faced during the pandemic, but also of the broader and longer-term nature of some of these challenges, the 74th World Health Assembly (WHA) in 2021 requested the WHO to develop, based on already existing normative documents of relevant international organisations (including WHO and International Labour Organization (ILO)), a global health and care worker compact providing guidance on how to ‘protect health and care workers and safeguard their rights, and to promote and ensure decent work, free from racial and all other forms of discrimination and a safe and enabling practice environment.’^37^ The 75th WHA in 2022 adopted a resolution calling on countries to ‘use, where relevant, the global health and care worker compact to inform national review, action and implementation to protect and support health and care workers.’^38^

This paper describes the approach and discusses the findings that led to the development of this compact, a milestone in the international health and care workforce policy domain.^39^ Importantly, the violations of the rights of health and care workers are anything but inevitable. From evidence-based recommendations from global organisations to policies and models from local to national levels—whether safe healthcare worker-to-patient staffing ratios, wide-ranging protections against discrimination and effective remedies, legal protections that are inclusive of often-excluded domestic care workers, or strong legal protections for whistle-blowers and unionisation, for example—effective, rights-respecting laws and policies exist, recommendations and models from which countries can learn, adapt, and implement.

## Methods

The care compact is deeply rooted in binding international law and further buttressed by non-binding international law and global policy frameworks. Based on the mandate given by the 74th WHA, we searched for existing legally binding treaties, along with non-binding resolutions, global strategies, and policy frameworks. The strategy entailed manually searching the instruments of relevant international bodies. Specifically, we reviewed five main sets of international instruments: (1) ILO conventions and recommendations (via the ILO’s Normlex Database, excluding any instruments that were not listed as being up to date); (2) WHO documents, such as strategies, resolutions, and policy guidance on human resources for health and occupational health and safety; (3) United Nations (UN) human rights treaties (via the Office of the UN High Commissioner for Human Rights list of core human rights treaties) and related documents; (4) UN General Assembly and Security Council resolutions; and (5) the Geneva Conventions and Additional Protocols, central to protections during armed conflicts. We also searched for declarations or similar instruments from major international health professional organisations, namely the World Medical Association (WMA), the International Council of Nurses, Public Services International, the World Federation of Public Health Associations, the International Confederation of Midwives, and the World Dental Federation.

In keeping with the mandate given by the WHA, documents were included if they had: (A) a legal (binding or non-binding) or other normative nature (such as conventions, resolutions, covenants and global strategies); (B) a global or regional focus and (C) a specific focus or specific provisions for protecting and safeguard health and care workers, or that covered all workers including health and care workers. We excluded document that: (A) had a purely or predominantly technical nature (such as policy briefs, technical guidelines, evidence reviews and opinion pieces) and no binding or prescriptive policy nature; (B) had only limited or unclear applicability to health and care workers; or (C) were specific to an individual country or jurisdiction.

In some instances, we conducted a selective search of secondary literature for areas that required additional information or support beyond what was available through international instruments we had identified, such as regarding whistle-blower protection and community health worker compensation.

We also had inputs from the chair of the Safeguarding Health in Conflict Coalition and interviewed the members of the International Year of Health and Care Workers Steering Committee, comprising experts and leaders from the following organisations and coalitions: the ILO; the OECD; the Frontline Health Worker Coalition; the Global Health Workforce Network; the International Council of Nurses; the International Pharmaceutical Federation; Public Services International; the Global Health Programme of the Health Service Executive, Ireland; and the WMA. Along with their own insights, many of these individuals also pointed us to specific documentation, which we also reviewed.

## Results

We included more than 70 documents on the basis of the inclusion and exclusion criteria described above. Many of the documents identified contain multiple relevant provisions and are therefore quoted more than once in the compact, with reference to different sections.

We categorised the legal and policy instruments identified according to four domains comprising 10 areas that collectively encompass the need to protect health and care workers from harm, providing supportive work environments, without discrimination, while empowering health and care workers to advocate for and safeguard their rights (table 1). Two domains relate to enabling work environment and working conditions for health and care workers (preventing harm and providing support), while the other two pertain to cross-cutting governance aspects (inclusivity through non-discrimination and empowering health and care workers through their essential labour rights).

**Table 1:** Compact domains and areas

Compact domain	Compact area	Key provisions	
Preventing harm	Dangers in health and care work environments	Implement occupational health and safety legislation and measures. Remove professional hazards, guaranteeing adequate personal protective equipment. Ensure safe staffing and working hours.	
	Health services for health and care worker	Ensure quality, accessible, affordable, confidential care for physical and mental needs. Offer diagnostic examinations, vaccination and other preventive care for occupational diseases and other health conditions.	
	Violence and harassment	Develop or strengthen laws, policies and strategies to prevent workplace violence and harassment. Enact requirements for employers to establish plans and actions to prevent violence and harassment. Ensure effective inspection, investigation, monitoring and enforcement mechanisms.	
	Attacks, particularly in fragile, conflict and violence situations	Ensure that laws are consistent with Geneva Conventions, including by reinforcing punishment for attacks on healthcare. Reinforce data collection, reporting and accountability.	
Inclusivity	Equal treatment and non-discrimination	Enact or strengthen antidiscrimination legislation. Ensure equal treatment and non-discrimination of health and care workers based on all UN treaty-recognised statuses, including as interpretted by treaty bodies. Ensure equal labour and human rights.	
Providing support to heath and care workers	Fair and equitable compensation	Review compensation and benefit structures to ensure fairness, equity and non-discrimination, present findings publicly, adjust government salary scales. Provide guidance and conduct audits to protect against unfair pay disparities and act to end discriminatory pay practices. Ensure that social protection policies extend to unpaid caregivers.	
	Social protection and non-financial support	Provide all health and care workers social security (health benefits for selves and families, disability and survivor benefits, paid maternity and parental leave, unemployment and old age benefits, and social security benefits when not working due to disability or child care). Establish national social protection floors.	
	Enabling work environments	Strengthen health systems to provide required equipment, supplies, medicines and other technologies. Establish/strengthen policy frameworks to facilitate enabling environments for all health and care workers. Review/update human resources for health strategies and policies and align budgets to assure sufficient resources for enabling environments.	
Empowering health and care workers to safeguard their rights	Right to freedom of association and collective bargaining	Legislate right to join and form associations without threat of retaliation. Enact penalties for interfering with right to associate. Enforce collective bargaining laws.	
	Whistle-blower protections and freedom from retaliation	Ensure protection for providing information on employer breach of statutory, legal regulatory or policy requirements. Review, develop and strengthen legal frameworks that safeguard whistleblowing rights. Educate health and care workers, employers and law enforcement on whistleblowing and non-retaliation rights.	

HCW, healthcare worker; UN, United Nations.

Each area is linked to human and labour rights, with specific references and provisions outlined in greater detail in the sections that follow.

### Preventing harm

#### Protection from occupational hazards

The International Covenant on Economic, Social and Cultural Rights (ICESCR) guarantees the right to safe and healthy working conditions as part of the right to just and favourable working conditions.^40^ Three ILO conventions (1981, 1985, and 2006)^41–43^ and recommendations^44–46^ align with this right in establishing international labour standards for occupational safety and health. These cover the material conditions of workplaces, training, protective clothing and equipment, assessment and surveillance of workplace risks, laws and policies, and risk reporting and monitoring systems.^41–43^ Another ILO recommendation specifically addresses nurses’ safe working conditions, including working time and rest periods.^47^ The WHO and ILO Framework for National Occupational Health Programmes for Health Workers provides extensive recommendations on ensuring occupational health and safety in the workplace.^48^

In addition to WHA resolutions 74.14 and 75.17, which specifically concern the global health and care worker compact, other WHA resolutions have also focused on workers’ health and safety, including WHA Resolution 60.26, which calls for essential health services and basic occupational care for all workers across all sectors,^49^ WHA Resolution 72.7, which focuses on safe and hygienic working conditions for health workers,^50^ and WHA Resolution 74.15, which calls on states to ensure nurses and midwives have the conditions and support required to safely practice.^51^ Meanwhile, the WHO Global Strategic Directions for Nursing and Midwifery recognise the need to ensure safe staffing.^52^ Other WHA resolutions aim to protect health workers against specific conditions, including hepatitis^53^ and polio,^54^ while WHA Resolution 73.1 calls on states to protect health and care workers against COVID-19, including through adequate PPE and psychosocial support.^55^ UN General Assembly Resolution 74/2 commits to scaling up healthier and safer workplaces and improving access to occupational health services.^56^

Other frameworks aim to ensure safety for workers with particular vulnerabilities or characteristics. The ILO Maternity Protection Convention^57^ and related Recommendation^58^ cover protections for workers who are pregnant or nursing, while UN General Assembly Resolution 75/156 stresses the importance of PPE and other protection, including hygiene and sanitary items and safe water, for female health workers, in addition to addressing their specific physical, mental, and psychological health needs and providing equal pay and freedom from violence.^59^

#### Health services for health and care workers

The right to health in the ICESCR includes a right to accessible, acceptable, and quality health services, as further specified by the UN Committee of Economic, Social and Cultural Rights’ General Comment 14.^15 40 60^ This requires health services for everyone, including health and care workers, while quality health services for the general population are only possible if health workers are protected from infectious diseases—such as through vaccination of health and care workers—that could harm patients.

Addressing workplace-related health harms, ILO’s Employment Injuries Benefits Convention provides for certain benefits for people with occupational injuries or diseases.^61^ A 1953 ILO recommendation outlines special considerations for medical care for workers employed in positions that involve a special risk to health—as is often the case for health and care workers.^62^ And WHA Resolution 74.15 calls for states to ensure mental health services for nurses and midwives.^51^

In 2015, WMA adopted a Statement on Physicians Well-being, which recommends that physicians’ well-being be supported within and outside the workplace^63^—a need for support that is relevant to all health and care workers.

Support for health and care workers has manifested in response to certain outbreaks, including HIV/AIDS, with UN General Assembly Resolution 60/262, which encompasses treatment,^64^ and COVID-19, with UN General Assembly Resolution 75/156, addressing physical and mental health needs for female frontline health workers,^59^ WHA Resolution 73.1, addressing safe testing, treatment, and palliative care for COVID-19, including for health and care workers,^55^ and WHO’s Call to Action: Vaccine Equity, calling for prioritising health and care workers globally for COVID-19 vaccination.^65^

#### Protection against violence and harassment

The ICSECR’s right to just and favourable working conditions includes the right to ‘freedom from violence and harassment,’ with the latter encompassing ‘physical and mental harassment, including sexual harassment.’^66^ The ILO Violence and Harassment Convention comprehensively addresses this right, establishing standards for protecting all workers and requiring states to adopt laws and policies for preventing and eliminating workplace violence and harassment, including by third parties. It also requires mechanisms for reporting and inspection, monitoring and enforcement, remedies for survivors, and training.^67^ UN General Assembly Resolution 74/2 commits to protecting health workers from all forms of violence, attacks, harassment, and discriminatory practices.^56^

Several instruments and documents address the right to and protection from violence and harassment for particular populations, including the Convention on the Rights of Persons with Disabilities,^68^ UN General Assembly Resolution 75/156, with respect to female health workers,^59^ and WHA Resolution 74.15, with respect to nurses and midwives^51^—though other particular marginalised populations, such as lesbian, gay, bisexual, transgender, queer or questioning, intersex, and other such (LGBTQI+) workers and those from ethnic minority populations, have limited specific protections.

Reaching beyond protection to support for health and care workers who experience workplace violence and harassment, WMA recommends support and counselling for health workers who experience or report violence^63^ —even as more, like ensuring reasonable accommodations to their work environment or schedule, may be required.

#### Protection against attacks in situations of fragility, conflict and violence

Prohibitions of attacks on health, including health workers, during armed conflicts is comprehensively addressed in international humanitarian law, which is grounded in the four Geneva Conventions (1949)^69–72^ and three Additional Protocols (1977, 2005).^73–75^

While the Geneva Conventions and Additional Protocols do not mention care workers, to the extent that they are engaged in health-related activities, they should be treated as health personnel, whom these instruments expressly protect. However, like all civilians, they are also protected from attacks under Geneva Convention IV and Additional Protocols I and II.^72–74^ The protection of health workers, as well as humanitarian workers, and civilians overall, and their infrastructure, has become part of customary international law, and is universally binding.^76–78^

UN Security Council Resolution 2286 demands compliance with international humanitarian law and international human rights law.^79^ UN Secretary-General recommendations on implementing the resolution and states’ international humanitarian law obligations include reinforcing legislative frameworks, promoting awareness and compliance with international humanitarian law, using available means to ensure that parties engaged in armed conflict respect their obligations, and contributing to data collection.^80^ And WHO, at the request of the WHA,^81^ established and maintains a database on attacks on healthcare.^82^

### Inclusivity

#### Equal treatment and non-discrimination

Several foundational human rights treaties guarantee the right to equal treatment and freedom from discrimination based on one’s status across numerous dimensions,^40 68 83–86^ including race, colour, national or ethnic origin, sex, sexual orientation, gender identity, disability, language, religion, marital and family status, and health status. Most of these treaties specifically provide for non-discrimination in the employment context,^40 66 68 84–86^ including specifically promoting equal employment opportunities and equal pay for equal work, among others.^68 85 86^

Several ILO conventions and recommendations establish universal labour standards in line with the right to non-discrimination, requiring policies to promote equal opportunity and treatment in employment,^87 88^ protect all workers with family responsibilities from discrimination,^89 90^ prevent discrimination based on HIV status,^91^ ensure equal treatment of migrant workers regarding key employment entitlements,^92–94^ and protect the health, job, and wage security of expectant and nursing workers.^57 58^

Also related to gender discrimination in light of the overwhelming predominance of women in the nursing professions, the ILO Nursing Personnel Convention provides that all nurses shall enjoy equivalent working conditions to other workers, including hours, compensation, leave entitlements, and social security.^95^ The ILO Domestic Workers Convention requires health and care workers in domestic settings to receive treatment equal to other such workers regarding hours, overtime compensation, rest and leave, minimum wage coverage, and remuneration, without discrimination based on sex.^96^

Long-standing WHA and UN General Assembly instruments require non-discrimination, including WHA Resolution 29.43, which also calls for expanding training, recruitment, and promotion for women in health work.^97^ WHA Resolution 74.15 calls for zero tolerance gender antidiscrimination policies and ‘establish(ing)’ senior leadership roles for nurses and midwives, through which they should have the authority to provide ‘input into health decision-making.’^51^ The UN 2030 Agenda for Sustainable Development urges states to end all forms of discrimination against women and adopt policies and legislation to promote gender equality.^98^

The WHO Global Code of Practice on the International Recruitment of Health Personnel requires equal work terms and conditions for migrant health workers as domestically trained health workers, including equal treatment in hiring, promotion, and remuneration.^99^

### Providing support

#### Fair and equitable compensation

Fair and equitable remuneration is part of the right to just and favourable working conditions, and expressly incorporated in human rights treaties,^40 66 68 84–86^ and includes not undervaluing any sector due to its gender composition.^66^ The ILO Equal Remuneration Convention proclaims the principle of equal remuneration for men and women for work of equal value,^100^ and the related Recommendation outlines measures to ensure equal remuneration, including equal access to vocational training, welfare, and job posts.^101^ UN General Assembly Resolution 75/156 encourages members to address the gender pay gap in the health.^59^

ILO conventions require migrant workers to be remunerated no less favourably than nationals,^92^ and domestic workers to receive overtime compensation equal to workers generally.^96^ The ILO Nursing Personnel Recommendation, specific to nurses but with generally applicable principles, calls for states to remunerate nurses commensurately with their qualifications, responsibilities, and experience, and to provide work supplies, clothing, and transportation without charge.^47^ Similarly, WHA Resolution 74.15 urges states to ensure fair remuneration of nurses and midwives,^51^ building on a call in WHA Resolution 63.7 for states to consult nurses and midwives on remuneration strategies.^102^

The Declaration of Astana declares that primary healthcare workers will be appropriately compensated in order to respond effectively population health needs.^103^ WHO guidance recommends that community health workers receive financial packages commensurate with their role demands, complexity, hours, and training.^104 105^

Both WHO and ILO highlight the need for transparency on actual remuneration levels to narrow and eliminate wage gaps,^9 106^ with these organisations and the OECD pointing to other necessary measures, including making avenues for recourse available to all health and care workers,^9^ strengthening wage legislation,^106^ and implementing gender-neutral occupational classification schemes,^107^ gender-transformative policies,^32^ and gender-equitable pay review processes.^9^

#### Social protection

Several ILO conventions and recommendations provide minimum standards for workers’ social security. The ILO Social Security (Minimum Standards) Convention covers medical care, sickness, injury, old age, unemployment, family, maternity, invalidity, and survivors’ benefits,^108^ with further guidance from a more recent ILO recommendation.^109^ Other ILO conventions and related recommendations from 1949,^88^ 1967,^110 111^ 1969,^112 113^ and 1975^94^ specifically cover non-discrimination in providing various benefits. The ILO Employment Injuries Benefits Convention^114^ and Recommendation^115^ include benefits for social service and hospital volunteers who are injured or become ill in the course of their work. Another ILO convention^95^ and related recommendation^47^ focus on nursing personnel and provide standards, including on maternity leave, sick leave, and social security.

Several instruments pertain to women and maternity protections. The ICESCR’s right to just and favourable working conditions includes protections for mothers during a reasonable period before and after childbirth, such as paid leave or leave with adequate social security benefits.^40^ Several ILO instruments also address maternity leave, as well as social security and childcare, regardless of workers’ gender,^57 58 116^ while CEDAW prohibits discriminating against women regarding social security benefits, including on grounds of pregnancy or maternity.^85^

#### Enabling work environments

Supportive work environments are closely tied to the rights to health and to just and favourable working conditions. Supportive supervision and opportunities for feedback create healthier and safer conditions for health and care workers and patients alike, while the stress of unsupportive environments can lead to mistakes, risking the health of workers and patients.

The ILO Nursing Personnel Convention requires policies on working conditions that help attract and retain nursing personnel,^95^ while the ILO Workers with Family Responsibilities Convention aims to protect workers with family obligations.^89^ WHA Resolution 64.6 calls for strategies for ‘safe and supportive working environments,’ particularly to increase the retention of health workers in rural areas.^117^ Towards the same end, WHO’s Global Strategy on Human Resources for Health Workforce 2030 and guidance on attracting and retaining health workers in rural areas focus on enabling work environments as a part of strategies to retain health and care workers in, or recruit them to, underserved areas.^2 35^

WHA Resolution 74.15 calls on states ‘to ensure that nurses and midwives are supported, protected, motivated, sufficiently aided, trained and equipped,’ and that they have ‘sufficient oversight and mentoring and…lifelong in-service training and further skills development.’^51^ WHO’s Global Strategic Directions for Nursing and Midwifery 2021–2025 provides further guidance.^52^ Similarly, the International Confederation of Midwives’ Bill of Rights for Women and Midwives recognises midwives’ right to an education to develop the skills and competency needed to remain and succeed in their profession.^118^ More generally, a WMA resolution calls for clear definitions of scope of practice and conditions for adequate support and supervision.^119^

### Safeguarding rights

#### Right to freedom of association and collective bargaining

All workers, including health and care workers, are entitled to free association and have the right to join and form trade unions, a right recognised independently and as a condition of just and favourable work,^40^ pursuant to several core human rights treaties.^40 68 83 84 86^ The ILO Declaration on Fundamental Principles and Rights at Work and several ILO conventions affirm these rights and set forth others.^120^ The Freedom of Association and Protection of the Right to Organise Convention provides for the rights for workers and employers to establish and join organisations.^121^ The Right to Organise and Collective Bargaining Convention states that workers shall be protected from antiunion discrimination and interference in their organisations, and requires states to establish machinery to protect workers’ right to organise.^122^ Additional ILO conventions and recommendations promote collective bargaining^123 124^ and extend the protections in the core conventions on the right to organise to workers’ representatives,^125 126^ public service personnel,^127 128^ and workers employed in domestic settings.^96^ Furthermore, the ILO promotes capacity-building for unions^9^ and social dialogue and the rights to unionise and collective bargaining as means for health and care workers to ensure their equal treatment and decent working conditions.^9 129^

#### Whistle-blower protections and freedom from retaliation

At least two treaties directly relevant to the compact expressly protect whistle-blowers: the ILO Violence and Harassment Convention (2019), providing protection for whistle-blowers regarding work-related violence and harassment,^67^ and the UN Convention Against Corruption (2004), protecting anyone who reports information on corruption to the competent authority.^130^ An ILO recommendation directs against taking measures prejudicial to workers for complaining of inadequate occupational health and safety protections.^44^ And the International Health Regulations (2005) enable WHO to protect the confidentiality of people who provide it with information on unusual public health events.^131^

The right to reveal information necessary to protect one’s own or the public’s health and safety is inherent in international human rights law. The freedom of expression includes ‘freedom to seek, receive and impart information and ideas of all kinds….’^83^ And such information advances the rights to the highest attainable standard of health and ‘safe and healthy working conditions.’^40^ Prohibiting health and care worker whistleblowing, or retaliating against such whistle-blowers, violates these rights.

Retaliation may come in another context as well: threats and harassment, arrest, torture, and even murder for impartially providing healthcare to political opposition figures, victims on the other side of an armed conflict, or alleged terrorists. This prohibition is firmly rooted in the Geneva Conventions and Additional Protocols^73 74^ and is contrary to states’ obligation to patients’ and health and care workers’ rights to life and health.^83 132^

More broadly, WMA declarations detail medical ethical responsibilities,^133–135^ which require impartial care and not participating in torture or other cruel, inhuman, or degrading treatment,^136^ and following ethical principles regarding medical research on human subjects.^137^ While speaking specifically to physicians, these declarations captures ethical responsibilities of all health and care workers.

## Discussion

Despite a wide variation in the scope, contents, and nature (such as health worker specific or broader), a robust international legal and policy framework exists on most aspects of health and care workforce employment and working conditions. Each area of the WHO global health and care workers compact includes specific human rights grounded in, and necessary to ensure, the right to health for all people. Beyond human rights treaties, other binding instruments, primarily ILO conventions, as well as non-binding instruments, including ILO recommendations, WHO guidance, WHA resolutions, and WMA declarations, ground the compact elements. Yet in many contexts across the world these commitments are not being fully met.

Fortunately, as noted earlier, while these commitments often go unmet, in some contexts governments and private employers are meeting their responsibilities, pointing the way ahead. With an empowering legal and policy environment—where civil and political freedoms are respected and health and care workers have a voice, and where governments are committed to human rights and labour rights through their own domestic legal and policy frameworks—along with political will and government recognition of the central role of health and care workers to the population’s health and well-being, these commitments are being realised.

Given the present disconnect between existing international legal requirements and practice in many contexts though, implementing the global health and care workers compact action areas will require creating more effective governance mechanisms, establishing new policies and effectively implementing these as well as those that have gone unimplemented in the past.

All this must be done in collaboration with health and care workers’ associations and unions, as well as representatives of health and care workers, including those serving marginalised and underserved communities. This participation is central to accountability and is, itself, a means of implementing health and care workers’ right to participate in the decision-making that affects their rights.^60^ It is also a means of implementing the Sustainable Development Goal target to ‘(e)nsure responsive, inclusive, participatory and representative decision-making at all levels.’^98^ Collaboration with civil society, such as through committees tasked with supporting governments to implement the care compact, is similarly critical.

A necessary early step towards this accountability and capacity to participate is ensuring that health and care workers, as well as populations at large, are aware of the existence and contents of the care compact. Likewise, as the compact recommends, health and care workers should be empowered with not only knowledge of the compact, but also with the skills needed to effectively engage policy-makers. Empowerment also entails enabling care workers, as well as community health workers and other health workers, who might not have formal organisations representing them to form such associations or unions.

One central value of the care compact lies in its potential to provide WHO Member States with a foundation to benchmark their national legislative and policy frameworks and to inform national review and implementation, in cooperation with health and care workers and their associations.

Implementation efforts could entail:

1. Reviewing the legal, regulatory, strategic, and policy frameworks and instruments in a jurisdiction to identify opportunities to revise them to incorporate recommendations in the compact.
2. Developing action plans at country, state/province, and district or other local levels to implement relevant compact recommendations.
3. Reviewing and revising monitoring and evaluation instruments and frameworks, including benchmarks and indicators, to ensure that they incorporate compact-relevant laws, regulations, and policies.
4. Allocating adequate resources for health and care workers to ensure appropriate, rights-fulfilling employment and working conditions.
5. Strengthening and empowering regulatory authorities to enable effective implementation of laws, regulations, and policies across public and private sectors.

These measures—and the compact provisions more broadly—will be more of a challenge in some countries than others. Lower-income countries in particular may be challenged in finding funds for certain provisions of the compact—ensuring fair pay for health and care workers, for instance. They may have limited capacity in their regulatory agencies, making effective enforcement of laws and policies, however themselves admirable, difficult. Limited human capacity may also impede the ability of government and health and care workers alike to identify models in other countries that they could adapt and incorporate into their own contexts.

Development cooperation, therefore, including through international technical and financial assistance, will be immensely valuable in some contexts to strengthen the capacity of countries to implement the recommendations in the care compact. It will be important to regularly review countries’ compact implementation, and to update the compact as emerging evidence and new normative and legal instruments may warrant.

The global health and care worker compact is an innovative approach to upholding the rights of health and care workers. It is the responsibility of countries, with the support of WHO and the international community more broadly, to join with health and care workers everywhere to enable the compact to achieve its potential.

## Data Availability

Data are available in a public, open access repository.
